# Improving Rebar Twist Prediction Exploiting Unified-Channel Attention-Based Image Restoration and Regression Techniques

**DOI:** 10.3390/s24144757

**Published:** 2024-07-22

**Authors:** Jong-Chan Park, Gun-Woo Kim

**Affiliations:** 1 Department of AI Convergence Engineering, Gyeongsang National University, Jinju 52828, Republic of Korea; pakw2022@gnu.ac.kr; 2 Department of Computer Science and Engineering, Gyeongsang National University, Jinju 52828, Republic of Korea

**Keywords:** Rebar Endpoint Prediction, image restoration, object detection, Unified-Channel Attention, pixel shuffle, regression

## Abstract

Recent research has made significant progress in automated unmanned systems utilizing Artificial Intelligence (AI)-based image processing to optimize the rebar manufacturing process and minimize defects such as twisting during production. Despite various studies, including those employing data augmentation through Generative Adversarial Networks (GANs), the performance of rebar twist prediction has been limited due to image quality degradation caused by environmental noise, such as insufficient image quality and inconsistent lighting conditions in rebar processing environments. To address these challenges, we propose a novel approach for real-time rebar twist prediction in manufacturing processes. Our method involves restoring low-quality grayscale images to high resolution and employing an object detection model to identify and track rebar endpoints. We then apply regression analysis to the coordinates obtained from the bounding boxes to estimate the error rate of the rebar endpoint positions, thereby determining the occurrence of twisting. To achieve this, we first developed a Unified-Channel Attention (UCA) module that is robust to changes in intensity and contrast for grayscale images. The UCA can be integrated into image restoration models to more accurately detect rebar endpoint characteristics in object detection models. Furthermore, we introduce a method for predicting the future positions of rebar endpoints using various linear and non-linear regression models. The predicted positions are used to calculate the error rate in rebar endpoint locations, determined by the distance between the actual and predicted positions, which is then used to classify the presence of rebar twisting. Our experimental results demonstrate that integrating the UCA module with our image restoration model significantly improved existing models in Peak Signal-to-Noise Ratio (PSNR) and Structural Similarity Index Measure (SSIM) metrics. Moreover, employing regression models to predict future rebar endpoint positions enhances the F1 score for twist prediction. As a result, our approach offers a practical solution for rapid defect detection in rebar manufacturing processes.

## 1. Introduction

The need for automated solutions in rebar manufacturing has been recognized for decades. Early efforts to optimize rebar manufacturing processes through computer-aided design and manufacturing began to emerge, addressing the limitations of traditional manual processes and laying the foundation for current smart production systems [1,2].

Recent advancements in Artificial Intelligence (AI) and robotics have significantly accelerated automation within the manufacturing industry. Deep learning, a crucial component of AI, plays a vital role in developing fully automated industrial AI systems for smart factories [3]. These systems aim to minimize defect rates in processes such as shaping rebar into various forms and constructing concrete foundations [4].

**Figure 1:**
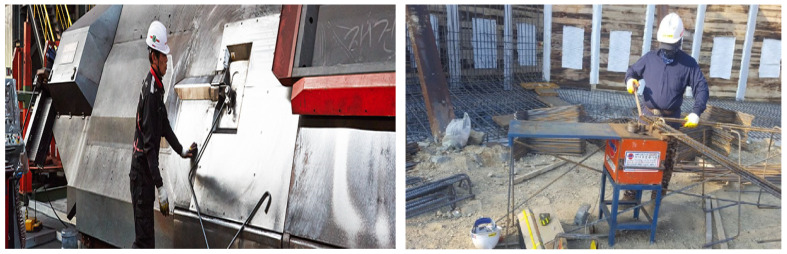
Traditional rebar processing site.

Despite these technological advancements, many rebar processing facilities still rely on traditional workflows. These conventional methods, as illustrated in Figure 1, typically involve using specialized equipment for cutting the rebar, followed by workers manually counting, packaging, and loading the rebar for delivery to construction sites. These manual processes heavily depend on the skill levels of workers to correct any production defects, which affects both the timeliness and accuracy of these corrections. Consequently, this traditional approach is not only time-consuming and costly but also raises potential concerns regarding quality and safety.

**Figure 2:**
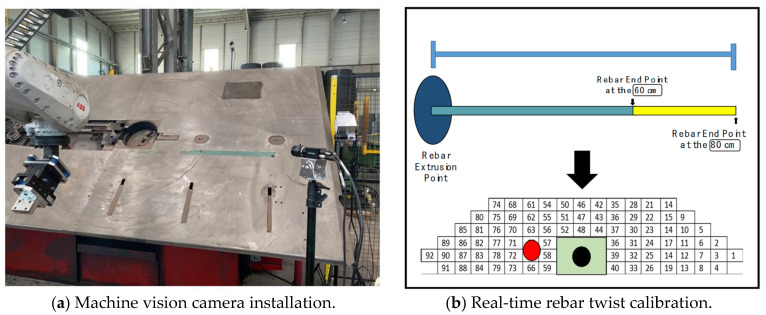
(**a**) shows the installation of a machine vision camera; (**b**) illustrates real-time calibration of rebar twist using grid cells. Detecting the rebar endpoint at the 60 cm mark from the extrusion point is crucial for predicting its future location at the 80 cm mark. Within the grid cell, black dots and green rectangular boundary boxes signify normal positions of rebar. Any deviation of rebar from this boundary box is marked by a red dot within grid cells. This deviation is indicative of twisting.

To address these issues, several studies have introduced automated smart production systems aimed at reducing processing defects and enhancing productivity through automated correction via real-time image processing. For instance, in the rebar processing workflow, it is crucial to detect rebar twisting in real-time, from the start of production until just before cutting. In the authors’ prior research [5], a machine vision camera was installed at the location where the rebar is extruded, as shown in Figure 2a, to assess the twisting of the rebar from after its extrusion until just before it is shaped. To analyze the captured images, a combination of algorithms was employed, including Oriented FAST and Rotated BRIEF (ORB), Region of Interest (ROI), and Hough Transform. These algorithms were utilized to differentiate the rebar from the background and to conduct feature matching, which determines the extent of the rebar’s twisting.

However, in actual manufacturing environments, the quality of images and lighting conditions significantly impacts the accuracy of image processing. In particular, under poor or irregular lighting conditions, the clarity and contrast of images can be reduced, leading to difficulties in object detection and feature extraction [6]. Adjustments to the machine vision camera’s focus distance, lens, and contrast brightness may not sufficiently counteract camera shakes or changes in lighting due to external factors, introducing environmental noise [7,8]. Consequently, existing image processing methods such as edge detection and background subtraction have limitations under these conditions. The effectiveness of edge detection can be compromised by low contrast between the rebar and its background or by noise and interference, while variable lighting conditions can cause background subtraction algorithms to misclassify image parts or fail to accurately isolate the rebar from its surroundings [9].

Super-Resolution Generative Adversarial Networks (SRGAN) are widely used to transform or restore low-resolution images to high-resolution images. This technique is currently utilized as a method for data augmentation and noise reduction in various fields, including medical imaging, satellite and aerial imagery, security cameras, disaster management, and the entertainment industry, to enhance the performance of AI-based systems when dealing with limited data [10,11,12,13,14,15]. However, not all information in the augmented images created by converting low-resolution images to high-resolution images with SRGAN significantly impacts the learning process of deep learning-based detection or classification models [16]. This is due to the potential partial loss of fine texture and essential information in the image or the excessive smoothing of the image’s characteristics. Additionally, in manufacturing environments, grayscale images are frequently used due to their processing speed and cost efficiency. However, since grayscale images rely solely on intensity variations to convey information, they are particularly sensitive to noise and often suffer from limited contrast [17]. This makes it difficult to distinguish between different objects or features within the image. Moreover, directly using them in deep learning models trained on RGB color images can lead to performance degradation. Therefore, enhancing SRGAN to include processing features that are robust to the characteristic contrast and intensity variations of grayscale images for specific areas can better preserve or highlight the structure and texture of those areas, contributing to improved performance of detection models.

Detecting rebar twisting through image processing is important. However, for increased accuracy, it is essential to precisely recognize the state of rebar twisting by using the coordinates of the dynamically extruded rebar endpoints, as illustrated in Figure 2b. More specifically, predicting the future coordinates of the rebar endpoints means that the state of rebar twisting can be more accurately represented, thereby reducing the prediction defect rate. Additionally, this method decreases the possibility of defects when determining rebar twisting based solely on image processing. However, due to the complexity of algorithms required to accurately track and predict the coordinates of dynamically extruded rebar endpoints in real-time within existing manufacturing processes, this method has not yet been fully automated or standardized.

In this paper, we propose a novel method designed to predict the future endpoint positions of rebar. This method aims to prevent potential twisting issues in rebar processing operations. The Unified-Channel Attention (UCA) and regression techniques developed for this purpose are capable of predicting the future endpoint positions of rebar with high accuracy. UCA emphasizes subtle differences in contrast and texture to efficiently extract crucial information from grayscale images, improving restoration and detection performance. Moreover, when dealing with large amounts of twisted and abnormal image data, issues such as bias are inevitably encountered during the training of object detection models. To address these challenges, we focus on rebar endpoints, extracting more information from existing small datasets of normal images and using them to convert low-quality grayscale images into high-resolution images. We then employ an object detection model to detect and track the endpoints of the rebar and apply regression analysis to the acquired coordinates to predict the error rate in the twisting of the rebar. The future positions of the rebar endpoints, as predicted through regression analysis, serve as a decisive indicator for determining the presence or absence of twists. The main contributions of this paper are as follows:

- **Unified-Channel Attention (UCA) Module:** This module combines average and max pooling to emphasize important contrast and texture features of grayscale images, enhancing the analysis and restoration of image brightness variations and dark area details. This improves the performance of object detection models.
- **Rebar Endpoint Prediction Through Linear and Non-Linear Regression Models:** We employ various linear and non-linear regression models, including Simple Linear Regression and Random Forest Regression, to predict future rebar endpoint locations using the central coordinates of bounding boxes extracted from an object detection model. During this process, the Euclidean distance is utilized to analyze the error rate in the positions of rebar endpoints based on the predicted and actual locations. This error rate is then used to classify the occurrence of rebar twisting, allowing for a more precise determination of the presence of defects in rebar.

The remainder of this paper is organized as follows. Section 2 provides a literature review of related work. Section 3 presents an overview and detailed methods of the proposed technique. Section 4 discusses our results and findings. Section 5 concludes the paper.

## 2. Related Work

Recent research has actively explored smart manufacturing systems that leverage cutting-edge AI technologies to automate and optimize manufacturing processes. These efforts focus primarily on the following areas:

**Model Development for Defect Recognition and Efficiency Enhancement:** Caggiano et al. [18] proposed using Bit-Stream Deep Convolutional Neural Networks (DCNN) for real-time defect recognition and classification during the Selective Laser Melting (SLM) process. Their model captures high-frequency features through dual streams and integrates low-frequency features with skip connections, minimizing information loss and enhancing classification accuracy. Kardovskyi et al. [19] developed an Artificial Intelligence Quality Inspection Model (AI-QIM) to automate rebar quality inspection in concrete structures. Utilizing Mask R-CNNs, for instance, segmentation and integration with a stereo vision camera, their AI-QIM effectively estimates rebar quantity, spacing, diameter, and length. Wang et al. [20] proposed an automatic rebar counting system using image processing and machine learning techniques. This system starts with image preprocessing tasks such as noise removal and edge detection from RGB images, followed by experiments with various machine learning algorithms, including Decision Trees, K-Nearest Neighbors (KNN), Support Vector Machines (SVM), traditional neural networks, and CNNs, with the CNN-based VGG19 model demonstrating the highest accuracy.

**Data Augmentation Techniques:** Given the complex and dynamic nature of manufacturing environments, characterized by variable lighting, movement, potential obstructions, and restricted access to certain areas for safety and efficiency, collecting high-quality training data is challenging. Tsai et al. [21] introduced CycleGAN for data augmentation in automated defect detection, generating and annotating defect images processed by U-Net to develop a Multi-scale Progressive Generative Adversarial Network (MAS-GAN) that improves surface defect detection efficiency and accuracy. MAS-GAN addresses the limitations posed by scarce surface defect samples and enhances detection model accuracy by generating images that closely mimic real ones. Yun et al. [22] discussed how data imbalance due to rare defects in the metal manufacturing industry, affects the generalization performance of Deep Convolutional Neural Networks (DCNNs). To overcome this, they proposed an automated defect inspection system using deep learning and data augmentation techniques. This system developed a new defect classification algorithm, Conditional CVAE (CCVAE), integrating Conditional Variational Autoencoders (CVAE) with DCNNs. The CCVAE effectively increases the quantity and diversity of training data by artificially generating and expanding learning data for various defect types, including rare ones, significantly improving the performance of DCNN models in experiments with real metal defect images, leading to superior performance.

**Image Restoration Techniques:** Research in image processing for converting low-resolution images to high-resolution as part of data augmentation has demonstrated multiple benefits. In manufacturing environments, where rapid data processing is crucial, low-resolution images are preferred due to their reduced storage demands and smaller size, which facilitate faster transmission. However, high-resolution images are more effective for in-depth image analysis and interpretation during model training. Image restoration techniques bridge this gap by enhancing low-resolution images to high-resolution, improving the quality of data augmentation, and boosting the overall performance of image-based models in these settings. Liu et al. [23] enhanced the Super-Resolution Generative Adversarial Network (SRGAN) by integrating a channel attention module to better capture high-frequency features and enhanced network performance by eliminating batch normalization layers. This reduction in computational complexity benefits tasks such as super-resolution and deblurring, thereby increasing network efficiency. They also refined the loss function to minimize the impact of noise, with their method surpassing other approaches in restoring high-frequency information and achieving higher Peak Signal-to-Noise Ratio (PSNR). In a related study, Liu et al. [24] proposed an efficient anomaly detection network named Skip-Attention GAN (SAGAN) for image-based anomaly detection. Traditional methods tend to overlook local anomalies by analyzing the overall difference between input and generated images, leading to unreliable detections. Their research introduced the skip-attention module to enhance the accuracy of the image’s latent representation and reduced the model’s parameter count by applying depth-wise separable convolution. Yang et al. [25] developed a Convolutional Block Attention Module (CBAM) based on SRGAN. Their attention module focuses on emphasizing important features while suppressing less critical ones, ensuring the quality of the network structure, and optimizing the generator network of SRGAN. Their experimental results showed the ability to train with fewer residual blocks, thereby reducing the reconstruction time.

**Object Detection Performance Enhancement:** Chen et al. [26] investigated the interaction between image restoration and object detection in underwater environments. They used visually enhanced data generated through a Filtering Restoration Scheme (FRS) and a Generative Adversarial Network Restoration Scheme (GAN-RS) to analyze the impact of these restoration techniques on object detection performance. Their evaluations using object detectors such as SSD and RetinaNet revealed that visual restoration plays a crucial role in reducing the domain difference between the training data and real underwater scenes. Liang et al. [27] introduced a Generalized Image Formation Model (GIFM) to address visual degradation in low-visibility environments. Unlike existing image restoration models, GIFM includes a light attenuation process within its new image formation model, showing outstanding performance in various tasks such as keypoint detection, object detection, and image segmentation.

Despite these advancements, several critical issues remain in the existing research on rebar manufacturing environments. These challenges significantly impede the effective detection and prevention of defects in the rebar production process, potentially resulting in suboptimal product quality, increased production costs, and safety hazards. Firstly, previous studies have predominantly focused on feature detection using traditional image processing techniques, which involve extracting features from edges, corners, ridges, or blobs before training deep learning models. However, these techniques may not effectively capture subtle defects and variations on rebar surfaces, often leading to missed detections or false positives. Moreover, there has been limited research on converting low-resolution images to high-resolution during manufacturing processes, which is crucial for accurate defect detection and analysis. Secondly, although our research includes detecting and tracking rebar endpoints using object detection models, these models are typically trained on RGB images that contain color information. In manufacturing processes, where image processing speed and efficiency are crucial, the use of grayscale images is advantageous. However, research on effectively extracting and learning features from grayscale images under varying brightness and contrast in manufacturing environments with irregular lighting is lacking. This gap affects the accurate detection and tracking of rebar endpoints, which is essential for identifying twists and ensuring product quality. Bui et al. [28] and Cho et al. [29] have conducted studies that underscore the significance of grayscale images in object recognition and detection. However, these approaches did not specifically address the challenges faced in rebar manufacturing environments or the necessity for real-time processing and adaptation to varying lighting conditions. Furthermore, despite advancements in image restoration and object detection techniques, these approaches face limitations in preemptively capturing product defects or errors during the manufacturing processes. While object detection systems can accurately recognize product defects or errors, it is crucial to track the coordinates of detected bounding boxes and analyze the prediction error rate to predict and respond to product defects or errors in advance.

To address the limitations identified in existing research, we propose a novel method that leverages image processing during the manufacturing process to overcome the constraints of image augmentation and manufacturing enhancement. Our research focuses on effectively extracting and analyzing contrast and texture features in grayscale images, aiming to develop a system capable of real-time processing that adapts to variations in lighting. This approach improves the accuracy of endpoint detection and twist detection in rebar manufacturing processes, facilitating precise quality control and classification. In this paper, we apply a UCA module, specifically designed for grayscale images, to the SRGAN to improve both image restoration and object detection performance. The UCA module integrates average and max pooling to highlight the subtle features of contrast and texture, enabling more effective extraction of essential information from grayscale images. This method not only captures and accentuates various characteristics of an image but also contributes to transforming low-resolution rebar images into high-resolution images. Furthermore, the enhanced images are used in object detection models, enabling more accurate detection of rebar endpoints. The bounding box coordinates obtained from this process are then analyzed using various linear and non-linear regression models, significantly reducing the prediction error rates. Our proposed method incorporates these regression models to predict the future positions of rebar endpoints based on the bounding box coordinates obtained from the object detection process. By analyzing the prediction error rate, our method can identify potential defects or errors before they occur, thereby enabling proactive quality control measures.

## 3. Proposed Method

**Figure 3:**
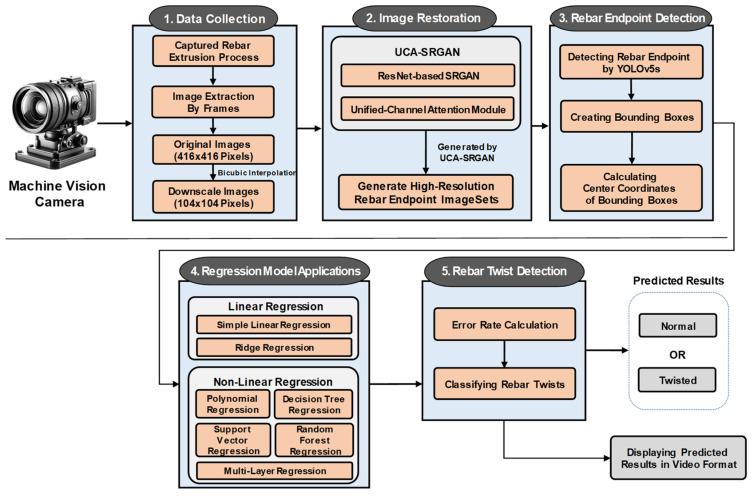
Overview of the proposed technique.

This section provides a detailed description of our proposed method, illustrated in Figure 3, which consists of five phases: (1) data collection, (2) image restoration, (3) rebar endpoint detection, (4) regression model application, and (5) rebar twist detection.

During the data collection phase, the rebar extrusion process is captured on video using a machine vision camera. The video is then decomposed into frames, and each frame is converted into an image. The extracted images are divided into two groups for image restoration: 1500 original rebar images and 1500 downscaled rebar images. In the image restoration phase, we apply the UCA module to the SRGAN based on ResNet to enhance the performance of rebar endpoint detection. This leads to the generation of high-resolution rebar endpoint image sets. The UCA-SRGAN model plays a crucial role in improving the quality of low-resolution grayscale images, which directly impacts the accuracy of subsequent phases. The third phase, rebar endpoint detection, involves selecting 500 high-resolution images from the generated rebar endpoint image sets to train the YOLOv5s model. The trained YOLOv5s model then detects rebar endpoints in real time by creating bounding boxes. Additionally, during this phase, the central coordinates of the bounding boxes are obtained in real time. The performance of this phase is significantly influenced by the quality of the restored images from the previous phase. During the regression model application phase, these central coordinates are used to train various linear and non-linear regression models, which then predict the future positions of the rebar endpoints every 60 frames. The accurate coordinates obtained from high-resolution images play a crucial role in enhancing the prediction performance of the regression model, thereby reducing the error rate in rebar twist detection. In the final phase, rebar twist detection, we calculate the error rate based on the distance between the actual and predicted rebar endpoint positions. We define a Rebar Twist as occurring when this error rate exceeds 5%. Specifically, if the normalized Euclidean distance between the predicted and actual rebar endpoint positions is greater than 5%, it is classified as a ‘*twist*’. Conversely, a result that falls within this 5% threshold is considered ‘*normal*’. The accuracy of this classification heavily depends on the precision of both the endpoint detection and the regression model predictions.

The interconnected nature of these phases is a crucial aspect of the proposed method. The high-quality images produced by the image restoration phase significantly enhance the accuracy of rebar endpoint detection. In turn, more accurate endpoint detection provides better input data for the regression models, leading to more precise predictions of future endpoint positions. These accurate predictions then contribute to more reliable twist detection. This sequential improvement in each phase contributes to the overall enhancement of the system’s performance in detecting rebar twists.

Furthermore, the results include predictions of rebar twists visualized in a video format, incorporating grid cells to detect twists by comparing the actual and predicted coordinates. Detailed explanations of each phase are provided in the subsequent subsections.

### 3.1. Dataset Collection

We installed a machine vision camera at the front of the rebar extrusion point and collected a total of 27 videos. These videos were captured under three different lighting conditions to assess the system’s performance across various environmental settings. The lighting conditions were as follows:

- **Standard brightness:** Brightness level of approximately 64,200 W LED light, exposure setting of 10 ms.
- **Medium brightness:** Brightness level of approximately 96,200 W LED light, exposure setting of 15 ms.
- **High brightness:** Brightness level of approximately 128,200 W LED light, exposure setting of 20 ms.

**Figure 4:**
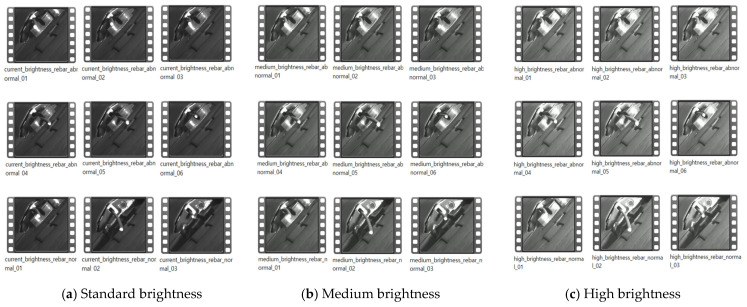
27 videos of the rebar extrusion process under different lighting conditions: (**a**) standard brightness (brightness level ~64,200 W LED, 10 ms exposure); (**b**) medium brightness (brightness level ~96,200 W LED, 15 ms exposure); (**c**) high brightness (brightness level ~128,200 W LED, 20 ms exposure). Each row within a lighting condition shows abnormal (**top** and **middle**) and normal (**bottom**) rebar extrusion processes.

As shown in Figure 4, (a) represents videos collected under standard brightness conditions, (b) under medium brightness, and (c) under high brightness. For each lighting condition, we collected three videos depicting normal rebar extrusion processes and six videos illustrating abnormal processes (i.e., twisting). This diverse collection of videos under various lighting conditions was used to identify limitations through experiments and to evaluate image restoration and detection performance across different lighting scenarios.

**Figure 5:**
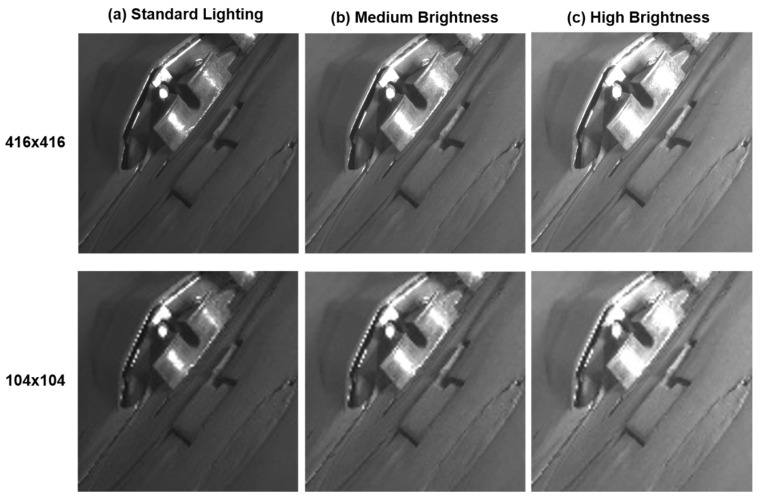
Image samples used in the restoration process: (**top**) 416 × 416 original high-resolution rebar images extracted from videos; (**bottom**) 104 × 104 low-resolution rebar images downscaled using Bicubic Interpolation.

During the image restoration phase, our goal is to use downscaled low-resolution images as input to restore them to their original high-resolution state. To achieve this, we selectively extracted 1500 original rebar images of 416 × 416 pixels from each set of 9 videos under different lighting conditions, as shown in Figure 5 (top). We then applied the Bicubic Interpolation technique to downscale these images to 104 × 104 pixels, creating 1500 low-resolution rebar images for each lighting condition, as shown in Figure 5 (bottom). This Bicubic Interpolation method interpolates the values of adjacent pixels, reducing the resolution of the images while smoothly maintaining the details.

We trained an image restoration model by linking these low-resolution rebar images with the original images as labels and evaluated the image restoration performance by comparing the restored high-resolution images with the original images.

### 3.2. Image Restoration

Our image restoration model leverages the ResNet architecture, incorporating several residual blocks to facilitate effective learning within deep neural networks. A key feature of this model is the integration of our proposed UCA module. The UCA-SRGAN model offers several key structural characteristics and advantages that contribute to its effectiveness in grayscale image restoration.

**Figure 6:**
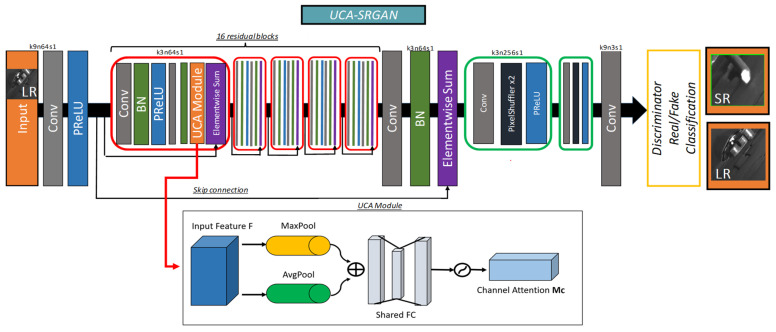
The overall architecture of UCA-SRGAN.

The UCA module efficiently utilizes channel information by combining average and max pooling operations, emphasizing important contrast and texture features crucial for accurate rebar endpoint detection. The model’s deep residual structure, comprising 16 residual blocks, mitigates the vanishing gradient problem and enables the learning of complex features essential for high-quality image restoration. To ensure smooth upsampling without artifacts, the model employs a pixel shuffle operation, efficiently transforming low-resolution feature maps into high-resolution outputs. The incorporation of a discriminator network in the adversarial training process encourages the generation of more realistic, high-resolution images, further enhancing the overall restoration quality. Figure 6 shows the overall architecture of our image restoration model, named UCA-SRGAN.

The first convolution layer of the generator (k9n64s1) employs a 9 × 9 kernel size, 64 filters, and a stride of 1 to extract initial features, particularly those related to detailed texture and contrast, from the input image. This layer is followed by 16 residual blocks, each consisting of two convolution layers (k3n64s1). These blocks enable the network to learn effectively without losing information in deep structures. Batch normalization and PReLU activation functions are applied between each convolution layer to enhance the network’s non-linear learning capabilities.

The UCA module operates on the feature maps extracted by the convolutional layers of the SRGAN. It performs channel-wise average and max pooling operations to capture the global and local importance of each feature channel. The AVG pooling identifies the overall intensity level of each channel, while the MAX pooling captures the most salient features. By considering both these aspects, the UCA module can effectively emphasize the important contrast and texture information in the input image. The pooled features are then concatenated and processed by two Fully Connected (FC) layers, which learn to assign channel-specific weights. These weights are applied to the original feature maps via element-wise multiplication, adaptively refining the feature representations. By employing ReLU and Sigmoid activation functions, the importance of each channel is adjusted and then applied to the original feature maps, resulting in the creation of the Channel Attention Map (Mc).

The refined feature maps are then passed through a series of upsampling blocks, which progressively increase the spatial resolution. Each upsampling block consists of a convolutional layer (k3n256s1), followed by a pixel shuffle operation. This operation rearranges the elements of a tensor to increase the spatial dimensions while reducing the channel dimensions, allowing for efficient and effective upsampling without introducing checkerboard artifacts. These blocks transform low-resolution images into high-resolution images using pixel shuffle operations and PReLU activation functions. The final convolution layer (k9n1s1) with a 9 × 9 kernel size, a single filter, and a stride of 1 generates the final high-resolution rebar endpoint images. This process plays a crucial role in restoring detailed and sophisticated high-resolution images. Lastly, a discriminator is employed to distinguish between the real and synthesized images, ensuring the authenticity of the restored images.

**Figure 7:**
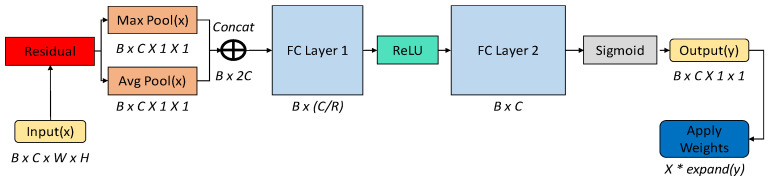
Architecture of Unified-Channel Attention (UCA) module.

Figure 7 illustrates the detailed architecture of the UCA module. Algorithm 1 describes the procedure for the UCA module.
**Algorithm 1.** UCA Module**Input:** input feature map *F* with dimensions (*B* × *C* × *W* × *H*) **Output:** channel attention map *Mc* with enhanced channel attention[**UCA Module Initialization**] **1:  initialize** *pooling operations*
**and**
*fully connected layers* **2:  define**
*Avg_Pooling*, *Max_Pooling* for spatial information aggregation **3:  define**
*FC_Layer1*, *FC_Layer2* for dimensionality reduction and expansion **4:  set**
*Activation (i.e.*, *ReLU*, *Sigmoid)* for non-linear transformation [**Feature Aggregation**] **5:  function** *aggregate_ feature(F):* **6:          for each**
*feature map in F*
**do:** **7:                apply**
*Avg_Pooling*, *Max_Pooling* **8:**                  *combined_features* 🡠 concatenate results of *Avg_Pooling* and *Max_Pooling* **9:                  return** *combined_features* **10:  end function** [**Channel Importance Weighting**] **11:  function** *compute_importance_weights(combined_features)*: **12:          initialize** *weights* array **13:          for each**
*set of combined_features*
**do:** **14:**                *reduced_features* 🡠 *FC_Layer1(combined_features)* **15:**                *activated_features* 🡠 *Activation(ReLU*, *reduced_features)* **16:**                *importance_weights* 🡠 *Activation(Sigmoid*, *FC_Layer2(activeted_features))* **17:                update** *weights* with *importance_weights* **18:          end for** **19:          return**
*weights* **20:  end function** [**Apply Weights to Feature Maps**] **21:  function** *apply_weights_to_features(F*, *weights):* **22:          for each**
*feature map F*
**and**
*corresponding weights*
**do:** **23:**                *adjusted_F* 🡠 *F* × *weights* **24:                update**
*Mc* with *adjusted_F* **25:          end for** **26:  end function** [**Main UCA Module Process**] **27:  initialize** input feature maps *F* with dimensions (*B*, *C*, *W*, *H*) **28:**  *combined_features* 🡠 *aggregate_ feature(F)* **29:**  *weights* 🡠 *compute_importance_weights(combined_features)* **30:**  *Mc* 🡠 *apply_weights_to_features(F*, *weights)* **31:  return** channel attention maps *Mc*

The input format is B × C × W × H, where B denotes the batch size, C is the number of channels (i.e., the number of feature maps), and W and H represent the width and height of the tensor, respectively. Average and max pooling are performed to calculate the average and maximum values for each channel, producing an output size of B × C × 1 × 1. These pooling mechanisms are essential for capturing the overall and distinct features of each channel, playing a critical role in estimating the brightness and darkness levels within each channel. The results from the average and max pooling are combined to form a vector of size B × 2C, which is then processed through two FC layers. The first FC layer reduces the dimensionality and applies the ReLU activation function, while the second layer normalizes the outputs to values between 0 and 1 using the sigmoid activation function. This process of applying weights to the feature maps involves multiplying the calculated importance weights by each channel of the input tensor ‘x’, adjusting the image according to the significance of each channel. This adjustment enhances the image quality by emphasizing important channels and suppressing less significant channels.

The integration of the UCA module is pivotal in effectively emphasizing and restoring key features in grayscale images. In grayscale images, where channel information is limited, accurately identifying and enhancing important information within each channel is crucial. Our UCA module addresses these challenges by identifying and adjusting the significance of each channel, thereby effectively highlighting important features of grayscale images. This enhances the accuracy of restoring fine textures and contrast. Additionally, the inclusion of residual blocks and pixel shuffle operations facilitates the effective learning of features in deep networks and a detailed transformation from low to high resolution. Our methods significantly improve the ability of the model to detect small or subtle changes in grayscale images and accurately restore details at higher resolutions.

### 3.3. Rebar Endpoint Detection

We employ the YOLOv5s model for the rapid and accurate detection of rebar endpoints. As the lightest version among the YOLOv5 models, YOLOv5s excels in the realm of real-time object detection. It provides fast processing speeds with high accuracy, making it particularly suitable for real-time object detection in manufacturing settings where computing resources are limited. The lightweight structure of YOLOv5s optimizes model efficiency, enabling swift inference times that are ideal for detecting rebar endpoints in real time.

**Figure 8:**
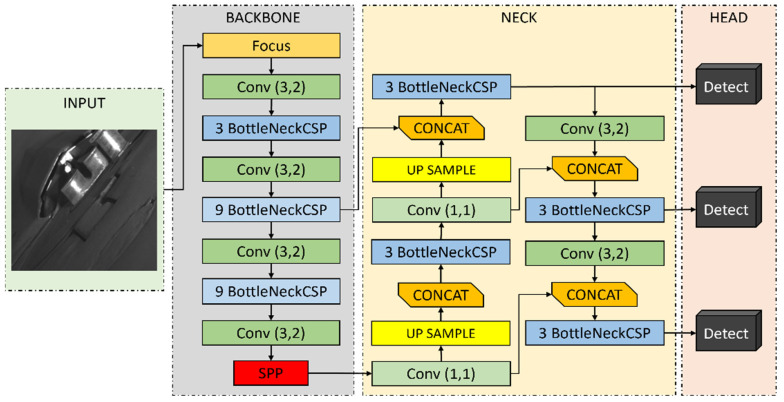
The architecture of YOLOv5s for detecting rebar endpoints.

The architecture of YOLOv5s is structured around three main components: Backbone, Neck, and Head. These components collectively play a vital role in the object detection process, directly influencing the overall functionality and performance of the model. Figure 8 illustrates the architecture of YOLOv5s as applied to rebar endpoint detection.

The Backbone, serving as the core component, extracts feature maps of various sizes from the input image. This extraction is achieved by dividing the image into four segments and combining them at the channel level, effectively reducing the resolution while increasing the density of critical information. It consists of several convolution and pooling layers, employing *BottleNeckCSP* and *Spatial Pyramid Pooling* (*SPP*) techniques. *BottleNeckCSP* enhances computational efficiency and gradient flow, thereby improving performance, while *SPP* boosts the spatial information of feature maps by pooling with various filter sizes. The *Focus* Layer, within the Backbone, serves as the basic feature extraction block, incorporating batch normalization and a leaky ReLU activation function. Following this, a *Convolutional Layer* (e.g., *Conv(3*,*2)*) with a 3 × 3 kernel reduces dimensions, and the initial *three BottleNeckCSP* layers enhance the model’s learning capabilities. After the second *Conv(3*,*2)*, *nine BottleNeckCSP* layers are arranged sequentially to further enhance complex pattern recognition. The *SPP* layer then extracts features of varying sizes, enabling the model to recognize objects across different scales.

The Neck effectively refines and reorganizes feature maps of various sizes by combining low-level and high-level features to enhance the accuracy and reliability of object detection. Following the Backbone’s *SPP*, a 1 × 1 convolution layer in the Neck increases the resolution of the feature maps through two upsampling processes, incorporating *three BottleNeckCSP* layers. This process, combined with intermediate output from the Backbone’s *nine BottleNeckCSP* layers via a *Concat* layer, integrates information and functional features from the Backbone, further refining the details. Additional operations involve *three BottleneckCSP*, *Conv(3*,*2)*, and *Concat* layers, setting the stage for the model’s Head to perform object detection functionalities.

The Head translates features from the Neck into final outputs, with object detection performed across three different *Detect* layers connected to the Neck’s *BottleNeckCSP* layers. Each *Detect* layer contains convolution layers that predict the bounding box parameters (*x*, *y*, *w*, *h*), object presence probability, and class probability. These parameters are essential for accurately determining the position, size, and class of objects. The bounding box parameters specify the object’s position and size, while the object and class probabilities indicate the likelihood of an object’s presence at that location and its classification, respectively.

After generating the bounding box for the detected rebar endpoints, we calculated the center coordinates (*x*, *y*) of the bounding box. The center coordinates of the bounding box are calculated as follows:

(1) $$Center(x,y)=\left(x_{1}+\frac{w}{2},\;y_{1}+\frac{h}{2}\right)\;$$

where $x_{1}$ and $y_{1}$ are the coordinates of the top-left corner, and w and h represent the width and height of the bounding box, respectively.

### 3.4. Regression Model Applications

In this phase, we utilized the central coordinates obtained from the object detection model to predict the future endpoint locations of the rebar using both linear and non-linear regression models. Although movements or location changes of objects within an image may initially appear straightforward, they often present complex challenges in actual manufacturing environments due to environmental noise from variations in image quality and lighting conditions. This complexity underscores the importance of selecting the appropriate regression model for each situation. For instance, while linear regression models may be susceptible to environmental noise, non-linear regression models offer enhanced robustness against specific types of noise and outliers.

**Figure 9:**
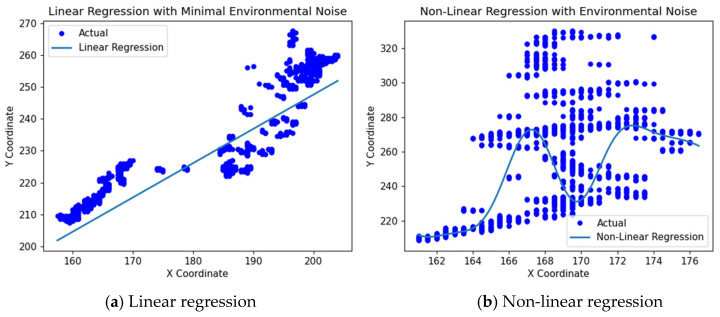
Application of linear and non-linear regression models for predicting rebar endpoint location: (**a**) linear regression applied to linear data with minimal environmental noise; (**b**) non-linear regression applied to non-linear data with environmental noise.

Figure 9 illustrates the application of linear and non-linear regression models for predicting the rebar endpoint location. Figure 9a depicts a linear regression model applied in scenarios with minimal environmental noise, visualizing both the actual and predicted locations of the rebar endpoint along the X- and Y-axes. Conversely, Figure 9b shows how a non-linear regression model effectively captures complex patterns and dynamic changes in data influenced by environmental noise. Although normal rebar extrusion typically exhibits a linear pattern, the endpoint of the rebar may exhibit slight shaking phenomena, adding non-linear characteristics. In contrast, abnormal extrusions reveal more non-linear patterns, increasing the uncertainty in prediction performance.

To address these challenges, we employed a variety of regression models to predict the future endpoint locations of the rebar, including Simple Linear Regression (SLR), Ridge Regression (RR), Lasso Regression (LR), Elastic Net (EN), Polynomial Regression (PR), Support Vector Regression (SVR), Decision Tree Regression (DTR), Random Forest Regression (RFR), and Multilayer Regression (MLR). These models, encompassing both linear and non-linear types, were selected to accommodate both normal and abnormal input images affected by environmental noise.

For each regression model, we used the central coordinates of the detected bounding boxes as input features and the corresponding future coordinates as target variables. Specifically, for a given frame t, we extracted the x and y coordinates of the rebar endpoints from the bounding boxes. These coordinates were then used to predict the y-coordinate of the endpoints in frame t+60, corresponding to approximately 2 s in the future. To ensure accurate parameter calibration and optimal hyperparameter selection for each regression model, we utilized GridSearchCV in our hyperparameter tuning process. To mitigate bias during model training, we randomly shuffled the training data and employed a stratified sampling strategy. The performance of each hyperparameter combination was evaluated using 5-fold cross-validation, with the mean squared error (MSE) as the scoring metric. In this process, the dataset was divided into five folds, with one fold serving as the validation set and the remaining four as the training set. This approach enables a comprehensive exploration of the hyperparameter combinations for each model through a rigorous 5-fold cross-validation process, repeated ten times. The combination yielding the lowest average MSE across the folds was selected as the optimal setting for each model. Table 1 lists the nine regressors we used, along with their respective hyperparameter settings.

### 3.5. Rebar Twist Detection

In this phase, we calculate the error rate based on the endpoint locations predicted by each regression model and select the model with the lowest error. Subsequently, we classify results that fall within a specific error rate threshold in the rebar manufacturing environment as ‘*Normal*’, while those exceeding thresholds are classified as ‘*Twist*’. To ensure accurate and reliable detection in real-time manufacturing environments, we define criteria for identifying Rebar Twist. The primary criterion for identifying a twist is an error rate exceeding 5%. This error rate is calculated as the normalized Euclidean distance between the predicted and actual positions of the rebar endpoint relative to the image’s diagonal length.

Initially, we calculated the error rate as the percentage obtained by normalizing the Euclidean distance between the predicted and actual positions relative to the diagonal length of the image. The error rate can be defined as follows:

(2) $$Error\;Rate=\left(\frac{\sqrt{{\left(x_{predicted}-x_{actual}\right)}^{2}+{\left(y_{predicted}-y_{actual}\right)}^{2}}}{\sqrt{{img\_width}^{2}+{img\_height}^{2}}}\right)\times 100\%$$

where *x_predicted_*, *y_predicted_* represent the predicted positions, and *x_actual_*, *y_actual_* denote the actual positions of the rebar endpoints within the grid boxes. The image width and height are represented by *img_width_*, *img_height_*, respectively.

We iteratively predicted the locations of the rebar endpoints using a set of linear and non-linear regression models, calculating the error rate for each prediction. This involves maintaining a cumulative error rate and counting the number of predictions to compute an average error rate for each model. The model with the lowest average error rate was identified as the best model. This model was then utilized for rebar twist detection, applying a predefined threshold. Algorithm 2 describes the procedure for rebar twist detection.
**Algorithm 2.** Rebar Twist Detection**Input:** dataset *D* containing (*x_actual_*, *y_actual_*, *img_width_*, *img_height_*), and a list of regression models *M*
**Output:** classification results indicating ‘*Normal*’ or ‘*Twist*’ and the best model *M*’ with the lowest error rate[**Calculate Error Rate**] **1:  function** *calc_error_rate(x_predicted_*, *y_predicted_*, *img_width_*, *img_height_):* **2:**        *error_rate* 🡠 calculate the prediction error rate with (*x_actual_*, *y_actual_*) **3:        return** *error_rate* **4:  end function** [**Model Selection**] **5:  define**
*model list M* (*M = {SLR*, *RR*, *LR*, *EN*, *PR*, *SVR*, *DTR*, *RFR*, *and MLR})* **6:  initialize**
*best model M*’ **as**
*None*
**and**
*lowest_error_rate*
**as**
*0.0* **7:  for each**
*model in M*
**do:** **8:        initialize**
*total_error_rate*
**as**
*0.0*
**and**
*count*
**as**
*0* **9:        for each**
*data point (x*,*y) in dataset D*
**do:** **10:**            *x_predicted_*, *y_predicted_* 🡠 predict using each model for data point **11:**            *error_rate* 🡠 **call function**
*calc_error_rate(x_predicted_*, *y_predicted_*, *img_width_*, *img_height_)* **12:**            *total_error_rate* 🡠 *total_error_rate + error_rate* **13:**            *count* 🡠 *count* + 1 **14:      end for** **15:**      *average_error_rate* 🡠 *total_error_rate/count* **16:      if**
*average_error_rate < lowest_error_rate*: **17:**          *lowest_error_rate* 🡠 *average_error_rate* **18:**          *best_model M*’ 🡠 *model* **19:  end for** [**Twist Classification**] **20:  if**
*lowest_error_rate ≤ 5%***:** **21:**      *classification* 🡠 ‘*Normal*’ **22:  else:**
**23:**      *classification* 🡠 ‘*Twist*’ **24:  return** *best model M*’, *classification*

This approach is designed to identify the most accurate model from a list of regression models for predicting the actual positions of rebar endpoints. The algorithm selects the model with the lowest average error rate for classification by evaluating the prediction error rate for each model against each dataset. A rebar condition is classified as ‘*Normal*’ if the lowest error rate is less than or equal to 5%; otherwise, it is considered a ‘*Twist*’.

**Figure 10:**
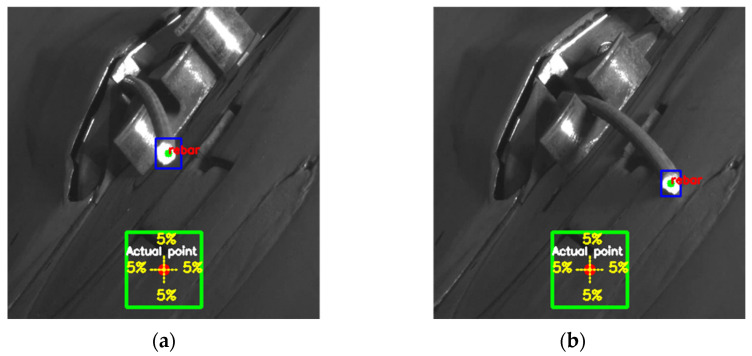
Visualization of grid cells for twist detection: (**a**) normal and (**b**) twist, displaying actual center coordinates and 5% threshold.

To effectively communicate the results, the findings are presented in a video format, utilizing grid cells to aid in rebar twist detection by contrasting the actual and predicted coordinates. Figure 10 illustrates the visualization of the grid cells for twist detection, displaying both the actual center coordinates and the predicted coordinates along with a 5% error threshold. In the normal rebar scenario (Figure 10a), there is a close match between the actual and predicted coordinates, with the error falling within the acceptable range. Conversely, in the case of twisted rebar (Figure 10b), there is a significant deviation between the actual and predicted coordinates, exceeding the 5% threshold, thereby triggering the twist detection mechanism.

## 4. Experiments and Results

In this section, we provide a comprehensive description of the performance evaluation of our proposed techniques. We introduce the metrics used to evaluate our methods and present a detailed discussion of the performance evaluation results. The performance of our techniques was evaluated through four experiments: (1) performance results of rebar twist detection, (2) threshold for the error rate parameter, (3) evaluation of the image restoration model’s performance, and (4) analysis of the rebar endpoint detection performance.

### 4.1. Evaluation Metrics

To evaluate the performance of rebar twist detection, we utilized four standard evaluation metrics commonly used in classification tasks: accuracy, precision, recall, and F1 score. These metrics were derived from the confusion matrix, which includes True Positives (TP), True Negatives (TN), False Positives (FP), and False Negatives (FN). Specifically, TP represents the number of instances where the model correctly predicts the presence of a twist in the rebar. TN refers to the number of instances where the model correctly identifies the absence of a twist, accurately recognizing rebar without any twisting. FP is the number of instances where the model incorrectly identifies non-twisted rebar as ‘*twist*’, representing a misclassification where the model predicts a twist in error. FN represents the number of instances where the model fails to detect a twist in the rebar, incorrectly labeling it as ‘*normal*’ when it actually contains a ‘*twist*’.

Accuracy indicates how often the model makes correct predictions out of all predictions and serves as a measure of the model’s overall performance. The equation for accuracy is defined as follows:

(3) $$Accuracy=\frac{TP+TN}{TP+TN+FP+FN}\;$$

Precision represents the proportion of instances predicted as ‘*twist*’ by the model that is actually ‘*twist*’. This metric reflects the model’s ability to avoid false positives. The equation for precision is defined as follows:

(4) $$Precision=\frac{TP}{TP+FP}\;$$

Recall represents the proportion of actual ‘*twist*’ instances that the model correctly identifies. This metric indicates the model’s ability to detect all positive instances. The equation for recall is defined as follows:

(5) $$Recall=\frac{TP}{TP+FN}\;$$

The F1 score is the harmonic mean of precision and recall and provides a balanced measure of the model’s performance. It considers both FP and FN, offering a comprehensive view of how effectively the model performs its predictions. The equation for the F1 score is defined as follows:

(6) $$F1Score=2\times \frac{Precision\times Recall}{Precision+Recall}$$

In addition to these classification metrics, we also employed the PSNR and the SSIM to evaluate the performance of our image restoration model. PSNR measures the ratio between the maximum possible power of a signal and the power of corrupting noise, expressed in decibels. A higher PSNR value indicates better image quality. The equation for PSNR is defined as follows:

(7) $$PSNR=10\;\cdot \;{log}_{10}\;\left(\frac{{MAX}^{2}}{MSE}\right)$$

where MAX represents the maximum possible pixel value in the image, and MSE represents the mean square error between the original and restored images.

SSIM evaluates the quality of an image by comparing its luminance, contrast, and structure with those of the original image. The SSIM values range from 0 to 1, with higher values indicating greater structural similarity between the original and restored images. The equation for SSIM is defined as follows:

(8) $$SSIM=\frac{\left(2_{\mu_{x}\mu_{y}}+C_{1}\right)\left(2_{\sigma_{xy}}+C_{2}\right)}{\left(\mu_{x}^{2}{+\mu }_{y}^{2}+C_{1}\right)\left(\sigma_{x}^{2}{+\sigma }_{y}^{2}+C_{2}\right)}$$

where x and y are the original and restored images, respectively; $\mu_{x}$ and $\mu_{y}$ are the means of x and y; $\sigma_{x}$ and $\sigma_{y}$ are the variances of x and y; $\sigma_{xy}$ is the covariance between x and y; and $C_{1}$ and $C_{2}$ are small constants to avoid instability when the denominator is close to zero.

### 4.2. Performance Results of Rebar Twist Detection

To evaluate the performance of our rebar twist detection technique, we conducted comparative experiments using various image processing and analysis techniques. These approaches were categorized into six groups: (1) traditional image processing, (2) low-resolution, (3) high-resolution, (4) enhanced high-resolution, (5) proposed enhancements, and (6) the proposed model.

For all comparison approaches, we first identified the rebar endpoints and obtained their coordinates. Then, we calculated the error rate, classifying an error rate of less than 5% as ‘*normal*’ and an error rate of more than 5% as ‘*twist*’. When using object detection to detect rebar endpoints, we employed YOLOv5s. Experiments were conducted for each approach, considering cases both with and without regression to predict rebar endpoint locations. When regression was used, we selected the model with the lowest average error rate from nine possible linear and non-linear regression models, such as Simple Linear Regression (SLR) and Support Vector Regression (SVR). When regression was not used, we calculated the alignment and orthogonality among the rebars to estimate the error rate at the rebar endpoint locations. To ensure robustness, we carried out an iterative evaluation for each approach. This evaluation involved 27 videos, each containing 30 frames, and was repeated 200 times across five sets, resulting in a total of 1000 iterations. Finally, we recorded and compared the average performance results for each approach.

Table 2 presents the performance results of the rebar twist detection. Proposed model indicates the highest-performing results and their corresponding scores.

**Figure 11:**
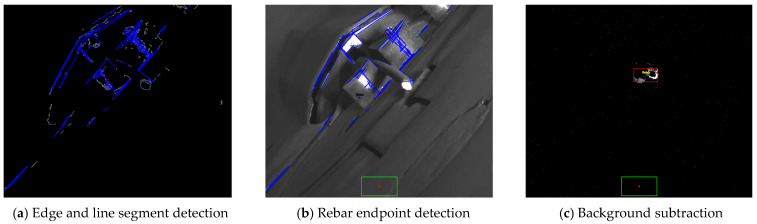
Results of traditional image processing techniques: (**a**) edge detection and line segment detection results based on Canny edge, (**b**) rebar endpoint detection results using Hough transform, (**c**) results of background subtraction and real-time tracking of center coordinates of moving area.

In the traditional image processing group, we evaluated the effectiveness of two existing approaches: edge detection + Hough transform based on the Canny edge and background subtraction + regression model (Gaussian mixture model). The edge detection + Hough transform approach, utilizing the Canny edge, exhibited the lowest performance metrics, with an accuracy of 0.3625, precision of 0.2664, recall of 0.1354, and F1 score of 0.1947. These results can be attributed to the method’s reliance on extracting edges from moving regions in the video and then detecting line segments using the Hough transform. However, the edge detection step often produces numerous irrelevant edges in the presence of lighting variations and background noise. Consequently, the Hough transform identified multiple line segments, some of which were mistakenly recognized as the endpoints of the rebar, leading to frequent misidentification of twisted rebars. Figure 11a,b illustrate how the detection of unnecessary line segments poses significant challenges to the precise identification of twist patterns in lighting-sensitive manufacturing environments, indicating that the edge detection + Hough transform approach is inadequate for handling the deformation of complex objects such as twisted rebars and fails to provide accurate central coordinates for the algorithm to effectively distinguish between twisted rebars. However, the background subtraction and regression model (Gaussian mixture model) approach demonstrated higher performance compared to the edge detection + Hough transform approach. It achieved an accuracy of 0.4892, a precision of 0.5110, a recall of 0.2156, and an F1 score of 0.3033. By incorporating the regression model, the error rate of the rebar endpoint location can be estimated more precisely, which contributes to improving the twist detection performance. As shown in Figure 11c, this approach effectively removed the background and accurately extracted the center coordinates of the moving area, which corresponded to the endpoint of the rebar. This enables more precise tracking of the endpoint location, facilitating improved twist detection performance. These results are particularly important for capturing the rebar movement in dynamic environments.

In the low-resolution group, we explored the impact of object detection and regression models on the performance of rebar twist detection using low-resolution images. This group served as a baseline to demonstrate the effectiveness of incorporating object detection and regression models compared with traditional image processing approaches. The low-resolution + object detection approach achieved an accuracy of 0.5775, precision of 0.5482, recall of 0.5073, and an F1 score of 0.5270. These results indicate that even with low-resolution images, employing object detection techniques can lead to improved performance compared to traditional image processing approaches. The object detection model can effectively localize the rebar endpoints, enabling better twist detection performance. Furthermore, when a regression model (Decision Tree) was incorporated alongside object detection, the performance of the low-resolution group further improved. The low-resolution + object detection + regression model showed an accuracy of 0.7172, precision of 0.7108, recall of 0.6846, and an F1 score of 0.6975. The integration of the regression model allows for more accurate prediction of the rebar endpoint positions, considering the spatial relationships and dependencies between the detected endpoints. However, while the low-resolution group demonstrates the benefits of object detection and regression models over traditional image processing approaches, their performance is still lower compared to high-resolution image restoration-based models. The characteristics of low-resolution images pose challenges in capturing fine details and accurately localizing the rebar endpoints, limiting the overall twist detection performance. This highlights the need to explore image restoration techniques to enhance the quality of low-resolution images before applying object detection and regression models, aiming to achieve even higher twist detection performance.

In the high-resolution group, we explored the impact of the image restoration technique, SRGAN, on rebar twist detection. Initially, we utilized SRGAN to enhance the spatial resolution of input images before applying object detection algorithms. We then evaluated the performance of SRGAN + Object Detection. The combination of SRGAN and object detection achieved an accuracy of 0.7960, precision of 0.7847, recall of 0.7447, and an F1 score of 0.7642, outperforming the traditional image processing and low-resolution approaches. Furthermore, we evaluated the combination of SRGAN + Object Detection with a regression model (Multi-Layer Regression (MLR)), which aimed to estimate the error rate at rebar endpoint locations more accurately. When MLR was used in conjunction with SRGAN and object detection, the performance further improved, with an accuracy of 0.8138, precision of 0.8437, recall of 0.7961, and an F1 score of 0.8192. These results demonstrate that the integration of object detection with super-resolution techniques can help capture spatial dependencies and improve the prediction of rebar endpoint locations. Moreover, incorporating a regression model enhances the ability to predict the future position of the rebar endpoint by considering its location variability, consequently leading to better twist detection performance. However, the existing simple detection methods for alignment and orthogonality among rebars, which estimate the error rate, cannot sufficiently predict the position of the rebar that continuously changes with high variability in real manufacturing environments. In contrast, applying a regression model accommodates this visual variability by modeling how the rebar’s position will change over time, enabling more accurate predictions of the future position of the rebar endpoint in a dynamically changing environment.

In the enhanced high-resolution group, we evaluated attention mechanisms to improve the quality of the image restoration technique. Attention mechanisms emphasize important features while suppressing irrelevant ones, ensuring the quality of the network structure. However, CBAM-SRGAN + Object Detection achieved a lower accuracy of 0.7766, precision of 0.7649, recall of 0.7045, and an F1 score of 0.7335 compared to SRGAN + Object Detection. This can be attributed to the limitations of channel attention in CBAM when dealing with grayscale images, as the amount of information that can be obtained through channel attention may be relatively limited compared to color images. Furthermore, spatial attention in grayscale images relies only on spatial characteristics such as texture and shape, which may not be sufficient for identifying important regions. The incorporation of a regression model with CBAM-SRGAN and object detection led to a decrease in performance compared to SRGAN + Object Detection + Regression Model (Multi-Layer Regression (MLR)). These findings underscore the importance of considering grayscale-specific approaches in improving the high-resolution image restoration process and their subsequent impact on twist detection performance.

The proposed enhancements group evaluated the approach using UCA-SRGAN + Object Detection, which achieved an accuracy of 0.8644, precision of 0.7983, recall of 0.9169, and an F1 score of 0.8535. The UCA module, specifically designed for grayscale images, was applied to SRGAN to improve image restoration and object detection performance. This approach proved to be particularly effective in extracting and learning features from grayscale images despite variations in brightness and contrast. Consequently, UCA-SRGAN can improve performance by effectively capturing and enhancing the relevant features in the rebar images, leading to improved object detection and twist detection performance without the need for regression modeling.

Finally, the proposed model group, comprising UCA-SRGAN for high-resolution image restoration, Object Detection for detecting rebar endpoints, and a regression model (Random Forest) for accurately estimating the error rate in rebar endpoint locations, achieved the highest performance among all the evaluated methods. It obtained an accuracy of 0.8829, a precision of 0.9376, a recall of 0.9029, and an F1 score of 0.9199. The UCA-SRGAN model enhances image quality and captures fine-grained details, while the object detection algorithm accurately localizes the rebar endpoints. The regression model, with its ability to handle complex non-linear relationships and its robustness to outliers, effectively estimates the error rate at the rebar endpoint locations. By leveraging the strengths of each component, the proposed model achieves significant improvements in accuracy, precision, recall, and F1 score for rebar twist detection, demonstrating its superior capability in this challenging application.

The experimental results demonstrate the effectiveness of our proposed approach in accurately detecting rebar twists in manufacturing processes. The integration of grayscale image restoration techniques, such as UCA-SRGAN, with object detection and regression modeling, significantly improves twist detection performance compared to traditional image processing techniques and standard super-resolution approaches. The attention mechanisms employed in UCA-SRGAN play a crucial role in enhancing the quality of the super-resolved images, leading to better object detection and twist detection performance. Furthermore, the incorporation of regression modeling helps estimate the error rate accurately at the rebar endpoint locations, contributing to the overall effectiveness of our proposed method.

### 4.3. Impact of Threshold for Error Rate

**Figure 12:**
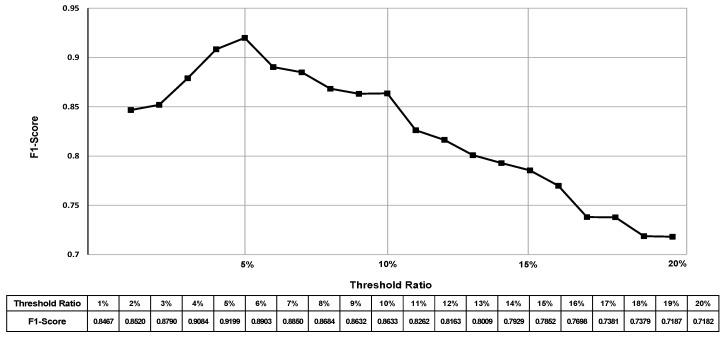
Change in F1 score according to threshold values ranging from 1% to 20%.

The values of the error rate threshold used for classifying rebar twists can significantly affect the performance of our proposed technique. In this experiment, we thoroughly analyzed the impact of threshold values on the detection performance using the F1 score. To determine the optimal threshold value, we varied the threshold from 1% to 20% in increments of 1% and evaluated the corresponding F1 score at each step. Figure 12 illustrates the impact of different threshold values on the performance of our rebar twist detection technique.

As shown in Figure 11, the F1 score demonstrates significant variance as the threshold for the error rate is varied. When the threshold value is set too low, such as 1% or 2%, our proposed technique becomes overly sensitive to minor deviations in the rebar shape, leading to a high number of false positives. As the threshold value increases from 1% to 5%, there is an initial improvement in the F1 score, suggesting that allowing a certain degree of tolerance towards error enhances the ability of our proposed technique to accurately classify twists. However, after 5%, further increasing the threshold value leads to a reduction in the F1 score. This reduction signifies a decline in the balance between precision and recall. As the threshold value increases, the model can begin to overlook actual twists, leading to an increase in false negatives, or it may misidentify regular rebars as twisted, resulting in an increase in false positives. Consequently, we have adopted this optimal 5% threshold for classifying rebar twists in our proposed technique. This threshold value enables our technique to accurately detect and classify rebar twists while minimizing false positives and false negatives.

### 4.4. Evaluation of the Image Restoration Model’s Performance

In this section, we compare the performance of our proposed model, combining the UCA module with SRGAN against three existing GAN and eight CNN-based image restoration models. The dataset used for training consisted of low-resolution grayscale rebar images, each with a size of 104 × 104 pixels. We evaluated the image restoration performance by comparing the restored high-resolution images with the corresponding original images, which have a size of 416 × 416 pixels. To ensure a fair comparison, the training parameters were set to a learning rate of 0.0002, a batch size of 1, and 500 epochs for all models. These images were collected by considering a diverse range of environmental noise, including camera shake and varying lighting conditions. To compare performance, we conducted a comprehensive analysis using PSNR and SSIM metrics. Additionally, we performed experiments to evaluate the image restoration models’ performance under medium and high-brightness lighting conditions.

Table 3 presents the comprehensive performance comparison of GAN and CNN-based image restoration models. Proposed model indicates the highest-performing results and their corresponding scores.

As shown in Table 3, the proposed UCA-SRGAN model consistently outperforms other models across all lighting conditions. Under standard brightness, it achieves the highest PSNR (40.5468) and SSIM (0.9716) scores. This superior performance is maintained under medium and high brightness conditions, although there is a general decrease in performance for all models as lighting intensity increases.

Analysis of the image restoration quality of the GAN-based model group revealed that the GAN model had the lowest PSNR and SSIM values. This indicates that the GAN models encountered difficulties in learning the distribution of grayscale rebar images, negatively impacting their image generation capabilities. The SRGAN model showed sufficient image restoration quality with a PSNR of 36.4418 and an SSIM of 0.9287, but its performance was relatively lower compared to the proposed UCA-SRGAN model. The CBAM + SRGAN model achieved a PSNR of 21.2176 and an SSIM of 0.9145, which is significantly lower than both SRGAN and UCA-SRGAN. The lower performance of SRGAN and CBAM + SRGAN can be attributed to their primary design focus on the restoration of color images. Color images inherently offer rich information on object boundaries, textures, and contrasts, which these models are optimized to exploit. In contrast, grayscale images lack these clear visual cues, making it more challenging for SRGAN and CBAM + SRGAN to distinguish between rebar and background areas effectively. Consequently, improving sharpness and detail in grayscale images becomes more difficult for these models, as they rely heavily on color information to guide the restoration process.

On the other hand, the proposed UCA-SRGAN model achieved the highest PSNR of 40.5468 and SSIM of 0.9716, demonstrating superior performance compared to other models within the group. This can be attributed to the UCA module’s effectiveness in emphasizing important features in grayscale images while suppressing irrelevant background noise. By focusing on these key characteristics, the UCA module enables the model to better capture and enhance the structure, texture, and contrast changes in the restored images. As a result, the UCA-SRGAN model is able to accurately detect rebar endpoints, even in the absence of color information.

In the CNN-based model group, ESPCN, RCAN, SRCNN, and DBPN show relatively high PSNR and SSIM scores, indicating their effectiveness in capturing local features and hierarchical representations of the input images. However, their performance is still lower than that of the UCA-SRGAN model. The EDSR, LapSRN, DRCN, and CARN models demonstrate moderate performance, with PSNR values ranging from 18.68 to 29.61 and SSIM values between 0.6135 and 0.8502. CNN-based models tackle the grayscale limitation by learning hierarchical features that capture the inherent structure and patterns of rebar images. These models employ deep convolutional layers to extract meaningful representations from the grayscale input, allowing them to restore missing details and improve image quality. However, the lack of color information may still affect their ability to precisely localize rebar endpoints, particularly in cases where the contrast between rebar and background is subtle. GAN-based models, particularly our proposed UCA-SRGAN, show better resilience to changing lighting conditions compared to CNN-based models. This is evidenced by the smaller performance drop in PSNR and SSIM scores across different lighting scenarios.

In the experiments, under medium brightness conditions, the ESPCN, RCAN, SRCNN, and DBPN models maintained relatively high scores in PSNR and SSIM but still performed somewhat lower than the UCA-SRGAN model. Overall performance decreased under medium brightness conditions, but the proposed UCA-SRGAN model still showed the highest performance. However, under high brightness conditions, the performance of all models significantly dropped. This can be attributed to the similarity in color between the rebar endpoints and background noise, leading to many false detections, as well as additional noise caused by flickering phenomena. This suggests that model performance can vary greatly depending on lighting conditions. Additionally, it was difficult to use these images for training YOLOv5, and additional noise occurred due to flickering during the process of collecting endpoint data under high brightness.

**Figure 13:**
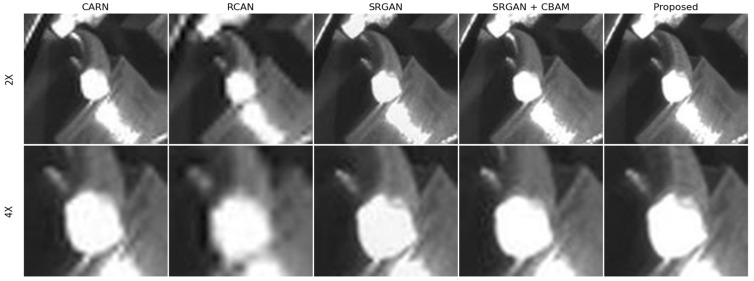
Comparative results of images generated by image restoration model: CARN, RCAN, SRGAN, CBAM + SRGAN, and the proposed model.

To further illustrate the qualitative differences between the models, Figure 13 presents visual comparisons of the restored images for rebar endpoints generated by five representative models: ESPCN, RCAN, SRGAN, CBAM-SRGAN, and the proposed UCA-SRGAN. For each model, images magnified by 2× and 4× are provided. The UCA-SRGAN model produces visually sharper and more detailed images compared to other models, with better preservation of rebar textures and edges. In the case of SRGAN and CBAM-SRGAN, blurry rebar patterns are observed when visually magnified. Interestingly, despite good quality metrics, images from the ESPCN and RCAN models sometimes appear to have lower perceptual quality to the human eye, with less defined edges and slightly fuzzy textures.

### 4.5. Analysis of Rebar Endpoint Detection Performance

In this section, we compare the object detection performance of YOLOv5s models trained on four different rebar image datasets: (1) low-resolution images, (2) original images, (3) images generated by SRGAN, and (4) images generated by our proposed UCA-SRGAN model. Each dataset consists of 1500 images, and the ratio of training and validation data was set to 8:2. To comprehensively evaluate the model’s performance under various lighting conditions, we conducted experiments under standard, medium, and high brightness settings.

To optimize the performance of the YOLOv5s models on these datasets, we carefully tuned both the model and data augmentation hyperparameters. The model hyperparameters, such as learning rate and number of epochs, directly influence the model’s training process and performance. To effectively calibrate the parameters and ensure the optimal selection of hyperparameters, we employed Optuna [30] in our hyperparameter tuning process. Optuna is an automatic hyperparameter optimization framework that efficiently searches for the best combination of hyperparameters, minimizing the time and effort required for manual tuning. Data augmentation hyperparameters control the augmentation techniques used to expand and diversify the training dataset. YOLOv5s relies on data augmentation to improve its robustness and prevent overfitting, especially when working with limited datasets. By applying transformations such as random rotations, flips, scale changes, and color variations to the training images, we can simulate various scenarios and enhance the model’s ability to detect rebar endpoints under different conditions. The hyperparameter values for data augmentation were empirically adapted, considering changes in lighting, scale, and orientation.

Table 4 and Table 5 present the YOLOv5s model hyperparameters and data augmentation hyperparameters used in our experiments, respectively.

The YOLOv5s models’ performance on each dataset was evaluated using precision-confidence and F1-confidence curves. These curves plot the precision and F1-score, respectively, against the confidence threshold used for detecting objects. By varying the confidence threshold, we can evaluate the model’s performance at different levels of detection certainty. In real-world manufacturing settings, these curves can be utilized in real-time video processing to enable accurate object detection under various environmental conditions, such as changes in lighting, machine movement, and orientation. Additionally, these curves can be used to find the optimal balance between precision and recall, minimizing false positives (i.e., incorrectly detecting non-rebar objects as rebar endpoints) and false negatives (i.e., failing to detect actual rebar endpoints) based on the specific requirements of the application. Table 6 presents the detection results for each dataset under standard, medium, and high brightness settings.

For the standard brightness, the low-resolution images dataset achieved a precision-confidence curve of 0.736 and an F1-confidence curve of 0.711, highlighting the challenges in accurate rebar endpoint detection. The low resolution and presence of noise hinder the model’s ability to distinguish between rebar endpoints and background. The original dataset showed slightly improved performance, with a precision-confidence curve of 0.793 and an F1-confidence curve of 0.742. The SRGAN dataset further enhanced the results, achieving 0.805 and 0.756, respectively. This improvement can be attributed to SRGAN’s attention mechanism, which helps focus on salient features of the rebar images. Our proposed model’s dataset demonstrated the highest performance, with a precision-confidence curve of 0.848 and an F1-confidence curve of 0.798. This significant improvement demonstrates the effectiveness of our proposed image restoration model. The UCA module in our model adaptively emphasizes important features in the grayscale rebar images while suppressing irrelevant background noise, resulting in restored images with enhanced structural details and clearer boundaries.

For the medium brightness, overall performance decreased compared to the standard brightness. The low-resolution medium dataset brightness achieved 0.602 and 0.554 for precision and F1 curves, respectively, showing a significant performance drop. The original medium brightness dataset improved slightly to 0.671 and 0.626 but still underperformed compared to the standard brightness. The SRGAN medium brightness dataset showed improved performance at 0.709 and 0.664 yet still fell short of the standard brightness results. Our proposed model’s medium brightness dataset achieved the highest performance in this category, with 0.741 and 0.693, but still showed decreased performance compared to the standard brightness.

For the high brightness, the performance degradation was even more pronounced. The low-resolution high-brightness dataset achieved 0.602 and 0.554 for precision and F1 curves. The original high brightness dataset slightly improved to 0.635 and 0.587. The SRGAN high brightness dataset showed better performance at 0.669 and 0.621 but still significantly underperformed compared to the standard brightness. Our proposed model’s high brightness dataset achieved 0.702 and 0.654, showing the highest performance in this category but still demonstrating a substantial decrease compared to the standard brightness results.

These results clearly demonstrate that performance degrades as lighting intensity increases (i.e., moving from standard to medium to high brightness datasets). This degradation is likely due to the increased difficulty in distinguishing between rebar and background in brighter images. Our proposed model shows the best performance across all brightness levels, particularly excelling with the standard brightness dataset. It effectively captures important features of rebar images while suppressing background noise.

**Figure 14:**
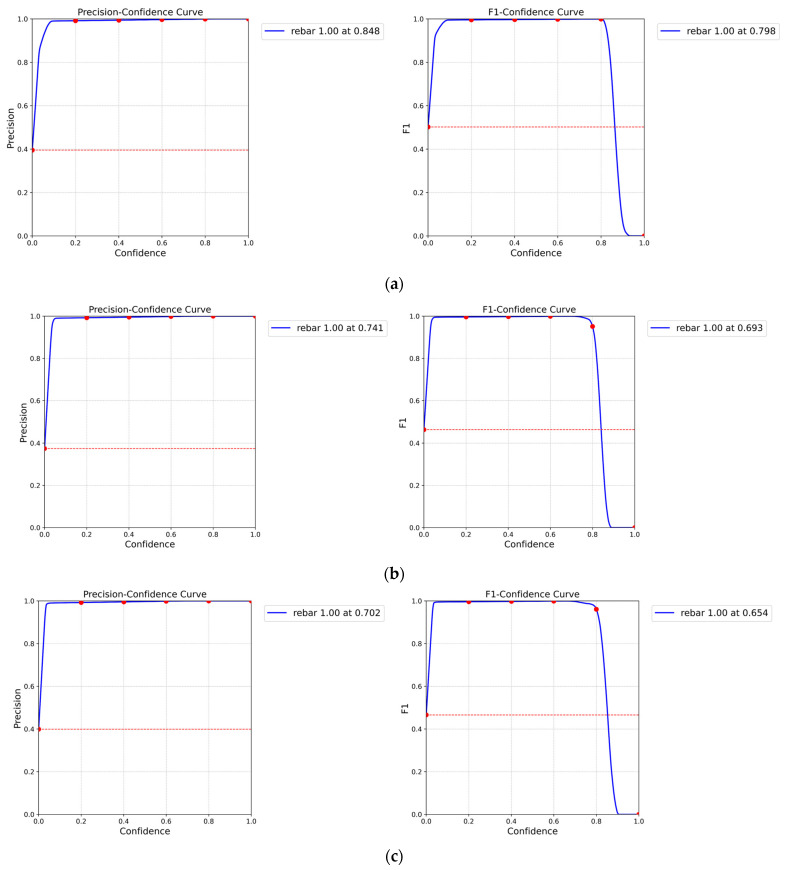
YOLOv5 training performance graphs for datasets generated by the proposed image restoration model: (**a**) Precision and F1 graphs for standard brightness resolution, (**b**) Precision and F1 graphs for medium brightness resolution, (**c**) Precision and F1 graphs for high brightness resolution.

Figure 14a–c show the rebar detection performance graphs of YOLOv5 trained on datasets generated by our proposed image restoration model for standard, medium, and high brightness, respectively. Notably, in all graphs, the F1 performance of the standard brightness dataset is superior when the confidence exceeds 0.8. This indicates that our proposed model performs best on the standard brightness dataset, effectively capturing crucial features of rebar images.

**Figure 15:**
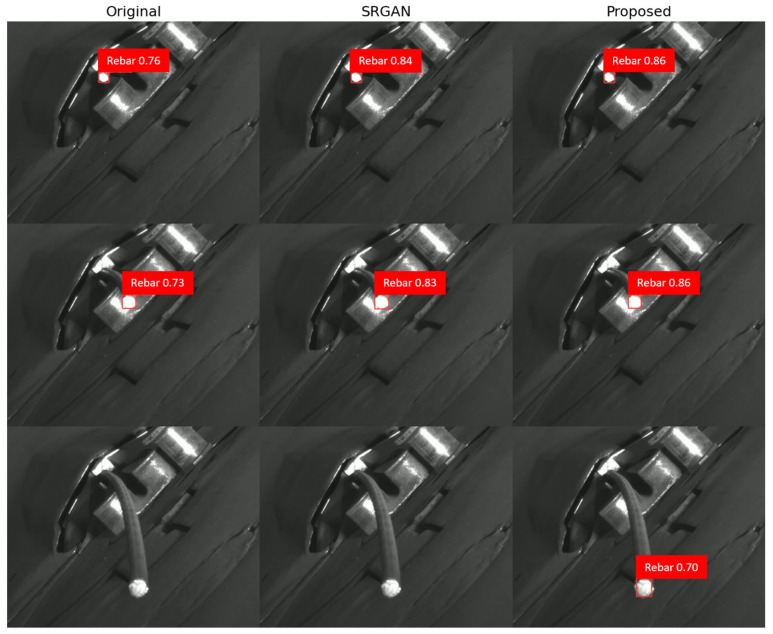
Visualizations of the detected rebar endpoints for each dataset.

To further validate the performance of our proposed model, we conducted real-time detection experiments using nine rebar videos under standard brightness conditions. Table 7 compares the average number of detections per frame and the average detection confidence for YOLOv5 models trained on three datasets: the SRGAN dataset, the Original dataset with contrast adjustment (Original + DA), and our proposed image restoration model dataset. Figure 15 presents visualizations of the detected rebar endpoints for each dataset under standard brightness conditions.

The SRGAN dataset achieves an average of 380 detections per frame with an average detection confidence of 0.6708. The Original + DA dataset improves upon this, with an average of 408 detections per frame and an average detection confidence of 0.7582. However, our proposed model dataset outperforms both, achieving an average of 410 detections per frame with an average detection confidence of 0.7748. The higher detection confidence indicates that our model is more certain about its predictions, reducing the likelihood of false positives and false negatives.

We observed that as time progressed, the confidence values of the predicted bounding boxes tended to decrease. As shown in Figure 15, when selecting only the bounding box with the highest confidence value above a 60% threshold, we found that for the original and SRGAN datasets, no bounding boxes met this criterion after a certain point, resulting in no detection. This leads to an increase in false negatives, where the model fails to detect actual rebar endpoints. This issue was not observed with our proposed model, which consistently maintained higher confidence values throughout the detection process. On average, the confidence values decreased by 0.05 per frame for the Original + DA dataset and 0.07 per frame for the SRGAN dataset, while our proposed model showed a decrease of only 0.02 per frame. This slower rate of decrease in confidence values for our model contributes to its superior performance in real-time detection tasks.

These real-time detection results demonstrate the practical applicability of our proposed image restoration model in enhancing the performance of rebar endpoint detection in manufacturing environments. By accurately restoring high-quality images from low-resolution and noisy inputs, our model enables the YOLOv5 object detector to achieve robust and reliable performance, even under challenging real-world conditions. The ability of our model to maintain higher confidence values over time further highlights its superiority in real-time detection tasks, ensuring consistent and accurate detection of rebar endpoints throughout the manufacturing process.

## 5. Conclusions

In this paper, we proposed a novel rebar twist detection technique that leverages high-resolution rebar images generated by a SRGAN integrated with a UCA module. Our approach accurately detects the center coordinates of the rebar using YOLOv5s and employs various regression models in real time to predict the future position of the rebar and determine the presence of twists. By experimentally comparing and analyzing several linear and non-linear models, we identified the model with the lowest error rate to maximize twist detection performance. This method can effectively respond to various types of data patterns, and by selecting an optimal model, the reliability and accuracy of the rebar twist detection process can be enhanced. The proposed system has the potential to significantly contribute to quality control and safety assessment in the construction and manufacturing industries by enabling more accurate identification of rebar twists.

However, our research has limitations that should be addressed in future work. One major limitation is the absence of experiments under varied lighting conditions and background noise levels, which could affect the system’s performance in real-world environments. To overcome this, we plan to develop a model that can dynamically adjust to different lighting conditions and thresholds. This will involve collecting a diverse dataset that encompasses a broad range of lighting conditions and background noise levels and training the model to adapt to these variations. Future work will also explore the application of advanced artificial intelligence learning methods, such as transfer learning and reinforcement learning, to further enhance the efficiency and performance of the system. Transfer learning can utilize knowledge from related tasks or domains to improve the accuracy and speed of rebar twist detection, while reinforcement learning can enable the system to adapt to changing environments and optimize its performance over time. We intend to conduct a series of experiments to assess the effectiveness of these methods and their impact on overall system performance. To ensure the practicality and applicability of the proposed system, we also plan to evaluate its performance at actual manufacturing sites for real-time processing optimization. This will involve conducting field tests and assessing the system’s performance in terms of processing speed, accuracy, and robustness, thereby providing a comprehensive validation of its capabilities in practical settings.

## Figures and Tables

**Table 1:** Candidate regressors and hyperparameter settings.

Type	Regressor Name	Parameter Setting
Linear	Simple Linear Regression (SLR)	None
	Ridge Regression (RR)	Alpha:1.0
	Lasso Regression (LR)	Alpha:1.0
	Elastic Net (EN)	Alpha:1.0, L1_ratio: 0.5
Non-linear	Polynomial Regression (PR)	Degree: 2
	Support Vector Regression (SVR)	C: 1.0, Epsilon:0.1, Kernel: Radial Basis Function (RBF), Polynomial, Sigmoid
	Decision Tree Regression (DTR)	Max_depth:None, Min_samples_split:2, Min_samples_leaf: 1
	Random Forest Regression (RFR)	N_estimators:100, Max_depth:None, N_jobs: −1, Min_sample_split: 2
	Multi-Layer Regression (MLR)	Hidden_layer_sizes:100, Activation: tanh, Solver: adam, Max_iter: 200

**Table 2:** Performance results of the rebar twist detection.

Group	Model	Accuracy	Precision	Recall	F1 Score
Traditional Image Processing	Edge Detection + Hough Transform (Canny Edge)	0.3625	0.2664	0.1354	0.1947
	Background Subtraction + Regression Model (Gaussian Mixture Model)	0.4892	0.5110	0.2156	0.3033
Low-Resolution	Low Resolution + Object Detection	0.5775	0.5482	0.5073	0.5270
	Low Resolution + Object Detection + Regression Model (Decision Tree (DT))	0.7172	0.7108	0.6846	0.6975
High-Resolution	SRGAN + Object Detection	0.7960	0.7847	0.7447	0.7642
	SRGAN + Object Detection + Regression Model (Multi-Layer Regression (MLR))	0.8138	0.8437	0.7961	0.8192
Enhanced High-Resolution	CBAM-SRGAN + Object Detection	0.7766	0.7649	0.7045	0.7335
	CBAM-SRGAN + Object Detection + Regression Model (Random Forest (RF))	0.8103	0.7097	0.7809	0.7436
Proposed Enhancements	UCA-SRGAN + Object Detection	0.8644	0.7983	0.9169	0.8535
Proposed Model	UCA-SRGAN + Object Detection + Regression Model (Random Forest (RF))	0.8829	0.9376	0.9029	0.9199

**Table 3:** Performance comparison of GAN and CNN-based image restoration models.

Group	Model	PSNR (Standard)	SSIM (Standard)	PSNR (Medium)	SSIM (Medium)	PSNR (High)	SSIM (High)
GAN-based models	GAN	3.9015	0.0778	3.8527	0.0752	2.1315	0.0636
	SRGAN	36.4418	0.9287	32.5474	0.9237	17.9010	0.8686
	CBAM + SRGAN	21.2176	0.9145	32.4397	0.9280	19.0736	0.8797
	Proposed Model (UCA + SRGAN)	40.5468	0.9716	34.9033	0.9425	29.8700	0.8878
CNN-based models	SRCNN	31.0370	0.8925	31.3183	0.8923	18.3161	0.8651
	ESPCN	33.2466	0.9278	19.9263	0.8563	19.1112	0.8296
	EDSR	21.3800	0.7261	19.8732	0.8782	19.0482	0.8565
	LapSRN	18.6787	0.6135	14.6029	0.4940	13.2134	0.4257
	DRCN	29.6144	0.8502	29.0567	0.8979	28.4577	0.8748
	DBPN	31.2165	0.9028	25.7865	0.8607	25.0529	0.8272
	CARN	21.4092	0.7636	19.9406	0.8797	19.1812	0.8564
	RCAN	33.8889	0.9368	29.2515	0.9005	28.8500	0.8792

**Table 4:** YOLOv5s training hyperparameters.

Hyperparameter	Value
Initial Learning Rate	0.01
Final OneCycleLR Learning Rate	0.2
Momentum	0.937
Weight Decay	0.0005
Warmup Epochs	3.0
IoU Training Threshold (IoU_t)	0.20
Anchor Multiple Threshold (anchor_t)	4.0

**Table 5:** YOLOv5s data augmentation hyperparameters.

Hyperparameter	Value
HSV-Hue Augmentation (hsv_h)	0.01
HSV-Saturation Augmentation (hsv_s)	0.7
HSV-Value Augmentation (hsv_v)	0.4
Horizontal Flip (flipir)	0.5
Mosaic	1.0
Adjust Contrast Gray	0.5

**Table 6:** YOLOv5s object detection performance results for each dataset under different brightness settings.

Dataset Type	Standard Brightness		Medium Brightness		High Brightness	
	Precision- Confidence	F1- Confidence	Precision- Confidence	F1- Confidence	Precision- Confidence	F1- Confidence
Low-Resolution	0.736	0.711	0.602	0.554	0.602	0.554
Original	0.793	0.742	0.671	0.626	0.635	0.587
SRGAN	0.805	0.756	0.709	0.664	0.669	0.621
Proposed Model	**0.848**	**0.798**	**0.741**	**0.693**	**0.702**	**0.654**

**Table 7:** Real-time YOLOv5 detection performance experiment results under standard brightness settings.

Dataset Type	Detection per Frame	Detection Confidence
Original + DA (Standard)	408	0.7582
SRGAN (Standard)	380	0.6708
Proposed Model (Standard)	410	0.7748

## Data Availability

The datasets used and/or analyzed during the current research are available from the corresponding author upon reasonable request.
